# Engineered Heart Tissue: A Novel Tool to Study the Ischemic Changes of the Heart *In Vitro*

**DOI:** 10.1371/journal.pone.0009275

**Published:** 2010-02-17

**Authors:** Rajesh G. Katare, Motonori Ando, Yoshihiko Kakinuma, Takayuki Sato

**Affiliations:** Department of Cardiovascular Control, Kochi Medical School, Nankoku, Japan; University of Milan-Bicocca, Italy

## Abstract

**Background:**

Understanding the basic mechanisms and prevention of any disease pattern lies mainly on development of a successful experimental model. Recently, engineered heart tissue (EHT) has been demonstrated to be a useful tool in experimental transplantation. Here, we demonstrate a novel function for the spontaneously contracting EHT as an experimental model in studying the acute ischemia-induced changes in vitro.

**Methodology/Principal Findings:**

EHT was constructed by mixing cardiomyocytes isolated from the neonatal rats and cultured in a ring-shaped scaffold for five days. This was followed by mechanical stretching of the EHT for another one week under incubation. Fully developed EHT was subjected to hypoxia with 1% O_2_ for 6 hours after treating them with cell protective agents such as cyclosporine A (CsA) and acetylcholine (ACh). During culture, EHT started to show spontaneous contractions that became more synchronous following mechanical stretching. This was confirmed by the increased expression of gap junctional protein connexin 43 and improved action potential recordings using an optical mapping system after mechanical stretching. When subjected to hypoxia, EHT demonstrated conduction defects, dephosphorylation of connexin-43, and down-regulation of cell survival proteins identical to the adult heart. These effects were inhibited by treating the EHT with cell protective agents.

**Conclusions/Significance:**

Under hypoxic conditions, the EHT responds similarly to the adult myocardium, thus making EHT a promising material for the study of cardiac functions in vitro.

## Introduction

Understanding the basic mechanisms and prevention of any disease pattern lies mainly on development of a successful experimental model. Tissue engineering is a newly developed technique that comprises of constructing a three dimensional structure from cardiomyocytes or progenitor cells and transplanting them in to in vivo reconstruction of the diseased myocardium [1], [2], [3], [4], [5], [6]. While all the studies have used EHT as a therapeutic tool, it not known if EHT can also replace the whole heart to study the characteristics of cardiovascular diseases in vitro, although Zimmermann and colleagues suggested that EHT could become a promising material to study cardiac functions in vitro [4]. Recent development of vascularized EHT [7], [8], [9] further supports our hypothesis that EHT could become a replacement for whole heart studies under in vitro circumstances. In this study, using advanced techniques of optical mapping along with other conventional techniques, we demonstrate that EHT responds similar to the whole heart under basal and stress conditions.

## Methods

One to three days old neonatal rats born to female Wistar rats (SLC, Japan) were used. All animals received humane care in compliance with the “Guide for the Care and Use of Laboratory Animals” prepared by the Institute of Laboratory Animal Resources and published by the National Institute of Health (NIH Publication No. 86–23, revised 1985) and approved by the ethical committee of Kochi Medical School, Japan.

### Cell Isolation

Cardiomyocytes were isolated from neonatal Wistar rats (postnatal day 1 to 3) by a fractionated DNase/Trypsin digestion protocol as described earlier [1]. The resulting cell population (50% cardiomoycytes/50% nonmyocytes [3]) was immediately subjected to EHT generation.

### Construction of EHT

**Figure 1:**
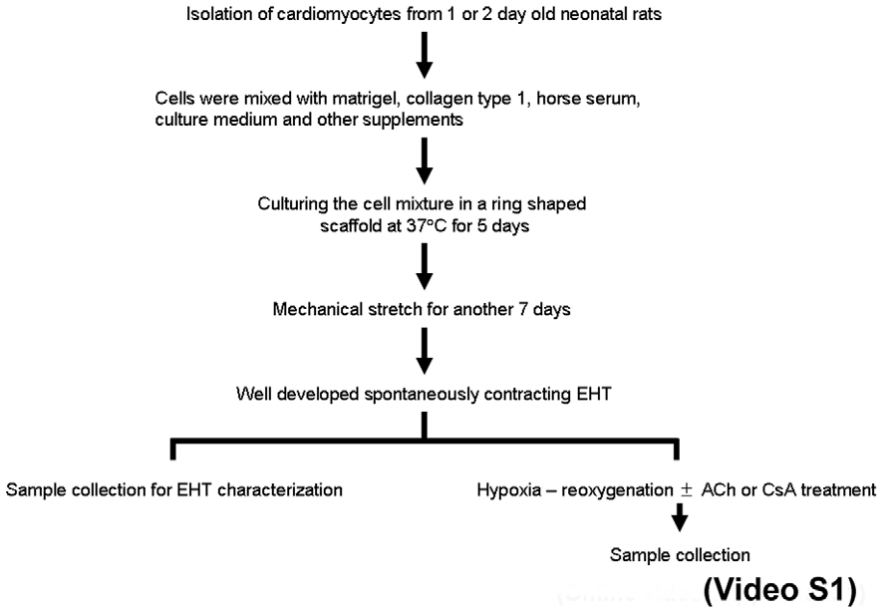
Experimental Protocol. Experimental protocol of the study.

EHTs were constructed as described previously. [4] Briefly, acetic acid solubilized collagen type I was mixed with concentrated culture medium (2× DMEM, 20% horse serum, 4% chick embryo extract, 200 U/mL penicillin, 200 µg/mL streptomycin). The pH was neutralized by titration with 0.1 N NaOH. Matrigel was added (10% v/v) if indicated. Finally, cells were added to the reconstitution mixture, which was thoroughly mixed before casting in circular molds (inner diameter, 5 mm; outer diameter, 10 mm; height, 5 mm). Within 3 to 5 days, EHT coalesced to form spontaneously contracting circular structures and were transferred on automated stretch devices conventionally constructed in our laboratory (**Fig 1** **and Video S1**).

### Hypoxia – Reoxygenation

To understand if fully developed EHT behaves similar to adult myocardium under stress, we used hypoxia-reoxygenation to simulate myocardial ischemia-reperfusion in vivo. For this purpose, the spontaneously contracting EHT was subjected to 6 h of hypoxia by culturing them with 1% O_2_ followed by 12 h of reoxygenation. At the end of experimental protocol the EHT was randomly assigned to undergo optical mapping to study the changes in conduction velocity or for protein extraction to study the changes in pro-survival signaling cascade.

To demonstrate if the EHT could exhibit similar responses of the adult heart to treatment with pharmacological agents under hypoxic stresses, the EHT was treated with cyclosporin (CsA, 0.2µM) or acetylcholine (ACh, 500µM) before hypoxia. We and others [10], [11], [12], [13] have previously demonstrated the cytoprotective effects of CsA or ACh on myocardium after acute ischemic injury (**Fig 1**).

### Optical Mapping

EHTs were superfused with warmed Tyrode's solution (135 mM NaCl, 5.4 mM KCl, 1.8 mM CaCl_2_, 1 mM MgCl_2_, 0.33 mM NaH_2_PO_4_, 5 mM HEPES, and 5 mM glucose) containing the voltage-sensitive dye di-4-ANEPPS (10 µM; Molecular Probes, Eugene, OR). After 7 min, the chamber was sealed and the dye was washed out as described earlier [14]. Action potentials (AP) were optically mapped using a CMOS-based high speed and high resolution optical mapping system (MICAM ULTIMA, Brainvision, Japan).

### Protein Preparation and Immunoblotting

As described previously [11], [15] the samples obtained at the end of experiments were prepared for immunoblot analysis. Extracted proteins were quantified with a BCA Assay Kit (Sigma). Equal amounts of proteins (50µg of total protein) were separated by SDS-PAGE, and transferred to a PVDF membrane (Millipore). After blocking nonspecific sites with 5% non-fat milk in TBS supplemented with 0.1% Tween20 over night, the membranes were probed with primary antibodies against Connexin 43 (1∶1000, Zymed Laboratories), Akt (1∶1000, Cell Signaling) and phospho Akt (1∶1000, Cell Signaling), BCl-2 (1∶1000, Cell Signaling), and α-sarcomeric actin (1∶1000, Abcam). Beta-actin (1∶1000, Cell Signaling) was used as loading control of the protein samples. Anti-rabbit IgG conjugated with horseradish peroxidase (diluted 1∶5000, Santa Cruz) was used as secondary antibodies and the membranes were finally developed with an ECL chemiluminescence reagent (Amersham). The samples were quantified by densitometry using Kodak Gel Logic 100 system (Kodak, Japan).

### Electron Microscopy

Following different protocols, the EHTs were divided into approximately 1 mm blocks and immediately fixed with cold 2% glutraldehyde. After 24 hr fixation at 4°C the samples were postfixed with 1% osmium tetroxide for 1 hr, dehydrated with increasing concentrations of alcohol (50%, 70%, 80%, 90%, and 100%; three times at each concentration) for 10 min each. Next, cells were infiltrated with propylene oxide for 15 min, followed by 1∶1 propylene oxide: epoxy resin for 4 hr. Samples were then embedded with fresh epoxy resin into molds and placed in an 80°C oven for 18 hr. Ultrathin sections were stained with uranyl acetate and lead citrate and were examined under an electron microscope [16].

### Statistical Analysis

Differences between two groups were analyzed using t-test (paired or unpaired as appropriate). Values are expressed as mean±SD. A P value of <0.05 was considered statistically significant for all parameters.

## Results

### Construction of Functionally Active EHTs

**Figure 2:**
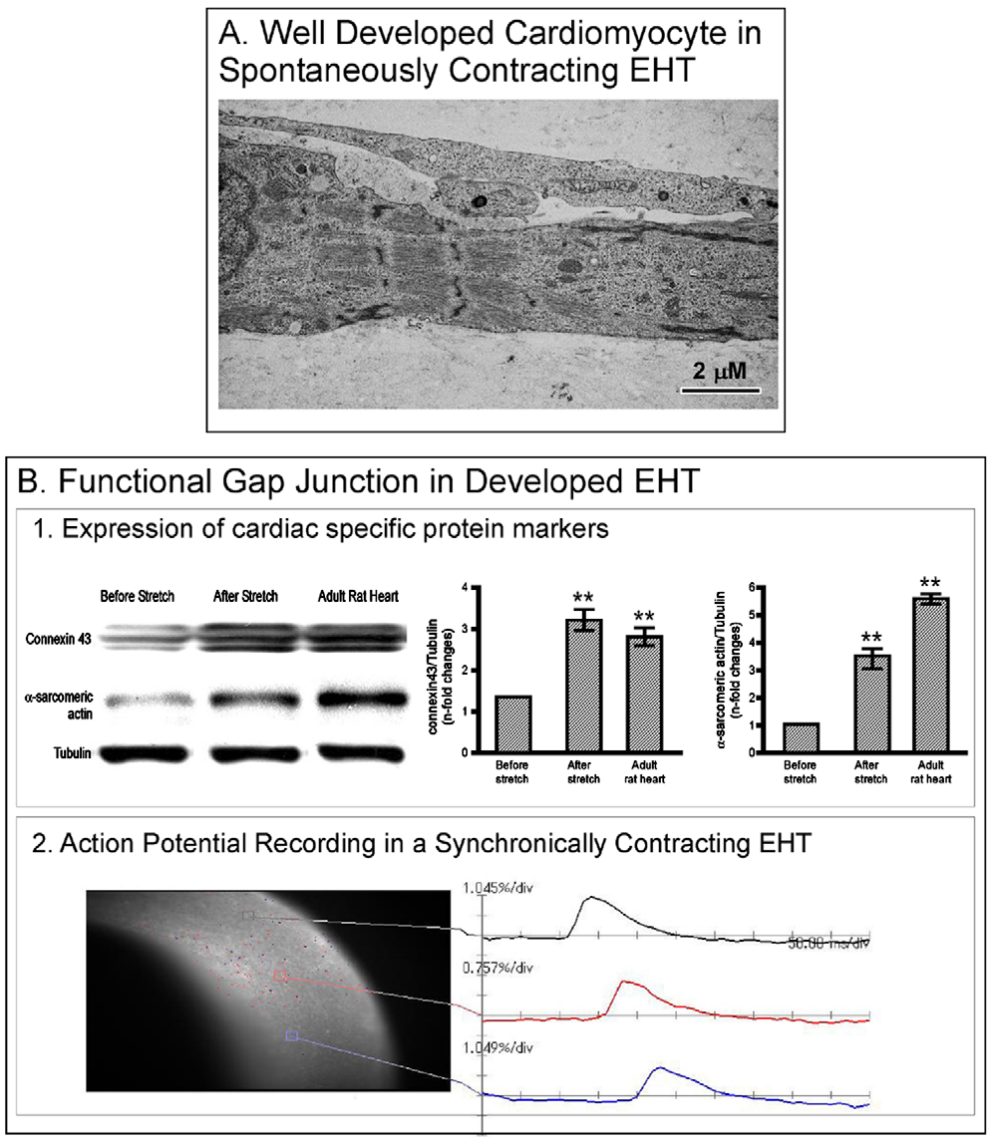
Characterization of the fully developed EHT. Samples were collected for electron microscopy (A) and western blotting (B1) after mechanical stretching. Tubulin was used as a loading control. Densitometry analysis was perfomed as explained in the methods. **P<0.001 versus before stretch. For optical mapping (B2), The EHT was loaded with voltage sensitive dye and images were captured with a CMOS-based high speed and high resolution optical mapping system.

Using the whole cell population from neonatal hearts we successfully constructed functionally active EHTs (**Video S1**). At day 5 after culture, the EHT demonstrated irregular fibrillation like contractions which became synchronous and regular after mechanical stretching (**Video S1**). The functionality of the constructed EHT was confirmed first using electron microscopy, showing fully developed adult cardiomyocytes with normally arranged sarcomeres (**Fig 2A**). Immunoblotting confirmed the expression of cardiac specific connexin 43 and α-sarcomeric actin (**Fig 2B**). Most importantly, mechanical stretches resulted in upregulation of these proteins (**Fig 2B**). Further, optical mapping showed synchronic conduction velocity as measured by the action potential recording across constructed EHTs (**Fig S1 and** **Fig 2C**).

### EHT Responds to Hypoxic Stresses

**Figure 3:**
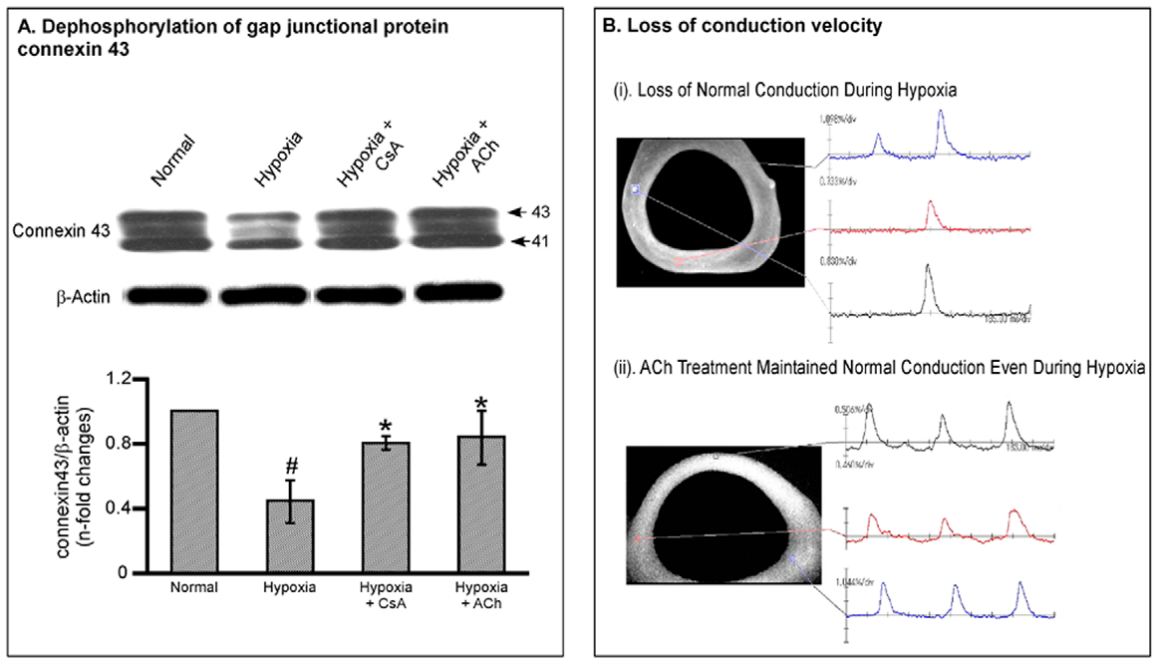
Response of EHT to hypoxic stress. **A.** Representative Immunoblotting analysis of connexin 43 in EHTs subjected to hypoxic stresses. Arrows indicate positions of phosphorylated isoform of connexin 43 (43 kDa) and nonphosphorylated isoform of connexin 43 (41 kDa) bands, respectively. Quantitative densitometric analysis represents the phosphorylated isoform of connexin 43. Values are mean ± SD ^#^P<0.05 versus normal group and ^*^P<0.05 versus hypoxic group. N = 5 in each group. **B.** Representative images showing the conduction defect evaluated by optical mapping, following exposure of EHTs to hypoxia, which was reverted by treatment with ACh. The synchronous conduction was lost in the EHT subjected to hypoxia. However, treatment with ACh prevented the conduction defect.

**Figure 4:**
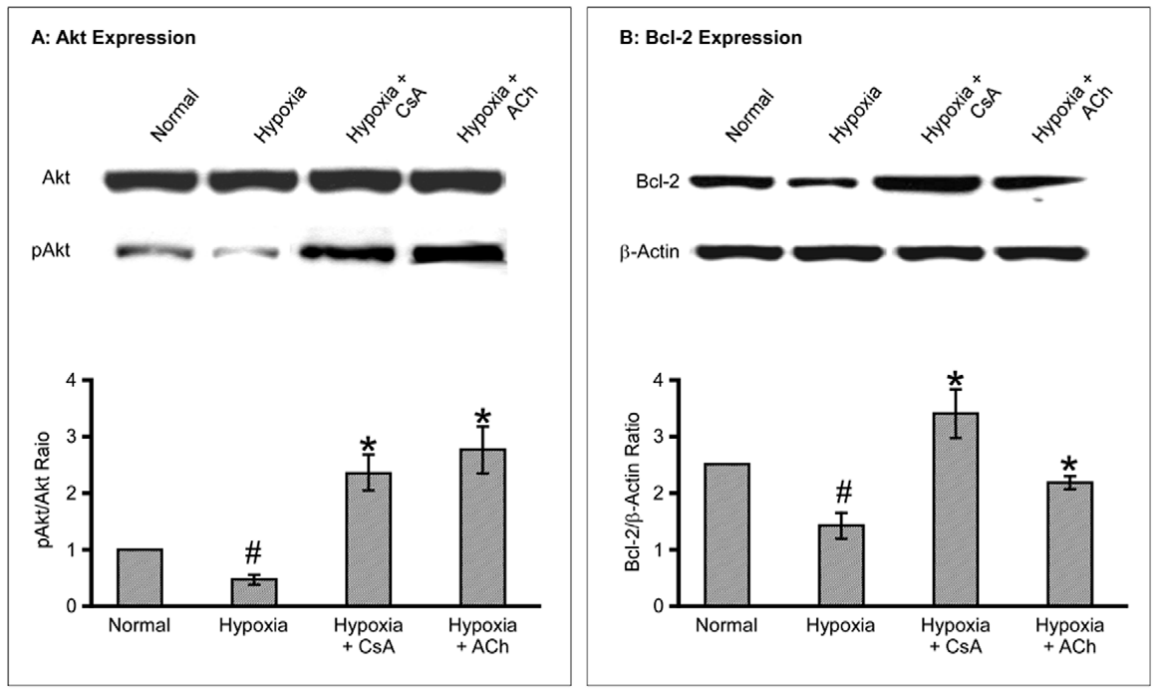
Cell survival Cascade analysis. Representative immunoblot and quantitative analysis of Akt (A) and Bcl-2 (B) in EHTs exposed to hypoxia. Values are mean ± SD ^#^P<0.05 versus normal group and ^*^P<0.05 versus hypoxic group. N = 5 in each group.

Next we tested if the constructed EHT could respond to hypoxic stress in a similar way to the whole heart. For this purpose, the EHTs were subjected to hypoxia and reoxygenation to simulate myocardial ischemia in vitro. Similar to the adult heart [12], hypoxia induced dephosphorylation of gap junctional protein connexin 43 (**Fig 3A**) and loss of normal conduction across EHT (**Fig 3B**). This was further confirmed by the molecular analysis of cell survival Akt and Bcl-2, both of which were downregulated in EHT subjected to hypoxia (**Fig 4**). Most importantly, treating EHT with pro-survival ACh or CsA [10], [16], markedly inhibited the hypoxia induced damage to the EHT (P<0.01, **Fig 3** **and** **4**).

## Discussion

Cardiac tissue engineering is an emerging field that may hold great promise for advancing the treatment of heart diseases [17]. EHTs have been developed in the view of myocardial replacement therapy [1], [2], [5], [8] and several studies have demonstrated the feasibility of EHTs in improving the cardiac function following myocardial injury [18], [19], [20]. However, to our knowledge this is the first study demonstrating the novel function of EHTs as a replacement model to the whole heart, for studying the response of the heart to any form of stresses, and to screen the pharmacological compounds for treatment of myocardial injury.

Understanding the basic mechanisms of diseases is accelerated by a good experimental model. Rodent models are widely used for the study of various cardiovascular diseases, especially to study the effect of long-term pharmacological interventions including long-term survival studies [21], [22]. However, apart from the outstanding cost, the use of animals needs expert skills and long time to yield reliable results. Moreover, the large number of animals are required to make reproducible results, especially in experiments involving pharmacological testing. However, as demonstrated in this study, the use of EHTs is easy, but at the same time, and does not compromise the quality of research outcome. From our experience it is possible to construct more than 5 pieces of EHTs from a single neonatal heart, which give the possibility to test the effect of different pharmacological agents on a single heart preparation. Futhermore, the EHT is useful in reproducing the effects of stresses and pharmacological agents on conduction velocity of action potentials, in a similar way to the whole heart. In addition, as demonstrated in the study, the survival signaling pathway in EHTs responds in a similar way to the whole heart under hypoxic stress.

Taken together, fully developed EHTs exhibit the characteristics of adult hearts and when subjected to hypoxia, they respond identical to the adult myocardium. Although, in vivo experiments are the golden standard for analysis of functional recovery following myocardial injury and pharmacological interventions, the developed EHTs could be used as a replacement for the adult heart in the situation of acute experimental setting, especially for studying the effects of stresses and treatment conduction velocity and molecular expressional changes.

## Supporting Information

Figure S1 Mechanical stretch synchronizes the contraction of engineered heart tissue.
(2.18 MB TIF)

Video S1 Video demonstrating the construction of engineered heart tissue.
(1.78 MB MP4)
