# Supplementary figures and images for: Engineered Heart Tissue: A Novel Tool to Study the Ischemic Changes of the Heart In Vitro
Source: PLoS One. 2010 Feb 17;5(2):e9275. doi: 10.1371/journal.pone.0009275 (PMC2822866; doi:10.1371/journal.pone.0009275)

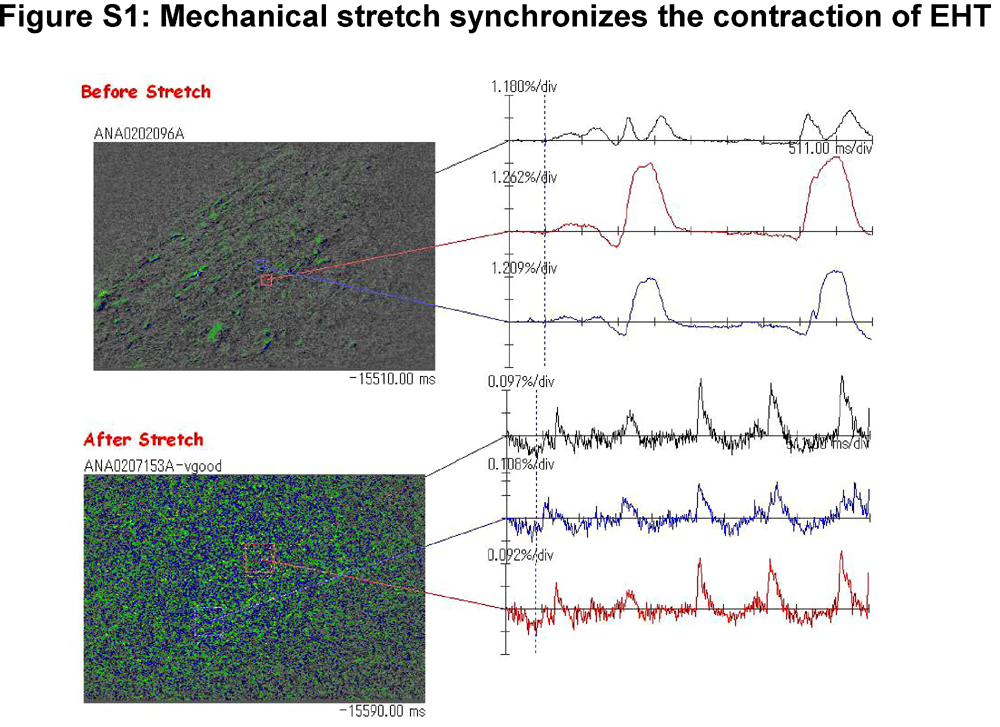

Supplement: Figure S1 — Mechanical stretch synchronizes the contraction of engineered heart tissue. (2.18 MB TIF) [file pone.0009275.s001.tif]
